# Immunohistochemical Review of Leydig Cell Lesions in Ochratoxin A-Treated Fischer Rats and Controls

**DOI:** 10.3390/toxins11080480

**Published:** 2019-08-20

**Authors:** Diana Herman, Peter Mantle

**Affiliations:** 1 Pathology Department, County Hospital Timisoara, Timisoara 300736, Romania; 2 Centre for Environmental Policy, Imperial College London, South Kensington, London SW7 2AZ, UK

**Keywords:** Leydig cell tumour, testicular cancer, ochratoxin A, immunohistochemistry, F344 rat, evidence-based diagnosis

## Abstract

Ochratoxin A is best known as a potent renal carcinogen in male rats and mice after necessarily protracted ingestion, although valid extrapolation to any human disease has not been verified. The hypothesis that the toxin is a cause of human testicular cancer was proposed a decade ago and has proliferated since, partly through incomplete study of the scientific literature. Archived tumorous rat testes were available from Fischer F344 rats exposed to continuous dietary exposure for half of or the whole life in London in the 2000s. Renal cancer occurred in some of these cases and testicular tumours were observed frequently, as expected, in both treated and untreated animals. Application of clinical immunohistochemistry has for the first time consistently diagnosed the testicular hypertrophy in toxin-treated rats as Leydig cell tumours. Comparison is made with similar analysis of tumorous testes from control (untreated) rats from U.S. National Toxicology Program studies, both of ochratoxin A (1989) and the more recent one on *Ginkgo biloba*. All have been found to have identical pathology as being of sex cord-stromal origin. Such are rare in humans, most being of germinal cell origin. The absence of experimental evidence of any specific rat testicular cellular pathology attributable to long-term dietary ochratoxin A exposure discredits any experimental animal evidence of testicular tumorigenicity. Thus, no epidemiological connection between ochratoxin A and the incidence of human testicular cancer can be justified scientifically.

## 1. Introduction

The carcinogenicity of ochratoxin A (OTA) in rats and mice was firmly established for the kidneys, particularly in males, in lifetime gavage exposure studies three decades ago [1,2], and since confirmed via dietary exposure [3,4,5]. At least nine months of exposure in the first year of life seems necessary for a high incidence of unilateral carcinoma [6,7]. Carcinogenic dose/response follows the classic semi-log pattern for which OTA has been accorded prime example status for carcinogenic toxicants [8]. The estimated threshold continuous daily lifetime exposure for male F344 rats was shown to be ~15 µg/kg b.w. in the NTP study and twice that for the Dark Agouti strain [6].

In the U.S. National Toxicology Program (NTP) toxicity study of OTA [2], the kidney was the only locus for primary cancer attributed to the toxin, from which distant metastasis was observed (e.g., in lung and abdominal serous surface nodules), but not in the testes. However, > 90% incidence of uni- or bilateral tumours was found in the testes of control (untreated) rats and in all treatment groups for OTA. The pathological descriptor was ‘interstitial cell, adenoma’ and thus no carcinogenic involvement of OTA was, or could have been, contemplated. Nor was any other adverse impact on well-being ascribed to the testicular tumours of the elderly Fischer rats exposed to OTA for most of their lifetime.

A hypothesis that OTA causes human testicular cancer was proposed [9] based on its natural occurrence as a food contaminant and as “a known genotoxic carcinogen in animals.” Testicular cancer was said to be of high incidence in Northern Europe, and was reported to be associated with subjects of high economic status and poor semen quality, all of which were seen to be generally associated with dietary exposure to OTA. Per capita human consumption data for coffee and pork were correlated with testicular cancer incidence across 20 countries worldwide. Most human testicular cancers were designated as being of germ cell origin (seminomas or non-seminomas [10,11,12]. Reference was made to rodent models for renal tumours [1,2], but there was no mention in the testicular dimension to these lifetime studies, particularly in [2], with the diagnosis of its testicular histopathology.

Since subsequent mouse experiments with exposure to OTA [13] give rise to OTA/DNA adducts in the testes, the hypothesis for humans was that foetal exposure during pregnancy induces lesions in testicular DNA and that puberty promotes these to testicular cancer. Unfortunately, in that publication, neither the absence of testicular cancer in mice given lifetime exposure to high-dose OTA (40 mg/Kg b.w.) while a 50% renal tumour incidence occurred [1], nor the ubiquity of tumorous testes in both control and OTA-treated rats of the NTP study [2], were explained. It is difficult to imagine how the authors could have been unaware of the two seminal toxicology studies focusing on OTA carcinoma in rodents. However, although the authors correctly noted the common embryology of the kidneys and testes from the mesonephros, this tissue also gives rise to the ovaries. Notably, primary ovary tumours were specifically absent from all control and OTA-treated female rats in the two-year NTP study [2]; in only one case did an ovary tumour occur, and this was attributed to metastasis from a kidney carcinoma, as has since been confirmed immunohistochemically [14].

Comments on the limitations of the testicular cancer hypothesis and its experimental exploration in mice [13] were subsequently made [15], since recognition of OTA/DNA adducts only indicates exposure to the toxin, as of course was implicit in the mouse embryo kidney by the experimental design. Apparent specificity for adducts in the kidney had not been tested against other organs, nor confirmed as widely distributed in circulating blood leucocytes. The present archived rat testicular tumours were offered for further study, but were not requested. Subsequent comment [16] repeats the contention that OTA “may be causally related to germ cell testicular tumors in mice and in men.” The present study will show that OTA/rat testicular tumours are not of germ cell origin, but are indeed interstitial cell adenomas, as historically described [2].

Following recent application of clinical immunohistochemistry to rat renal tumours caused by OTA exposure in a pilot study [14], we have here explored histopathology in testicular lesions from the same and experimentally associated animals recruited from several lifetime rat studies [4,5,7]. The objective has been to assess whether chronic OTA exposure had made even subtle changes to the testicular lesion phenotype, which appears to be a normal outcome of ageing in some laboratory rat strains, including the Fischer rats used extensively for many years in NTP studies. The approach has been objective clinical diagnosis without any preconceptions. Only subsequently have tumorous rat testes of untreated control examples from NTP Archives been included for comparison and for scientific rigour, although not to question their previous pathological diagnosis.

## 2. Results

### 2.1. Morphology

#### 2.1.1. OTA-Treated Rats

Findings for OTA-treated rats are summarized in Table 1.

**Figure 1:**
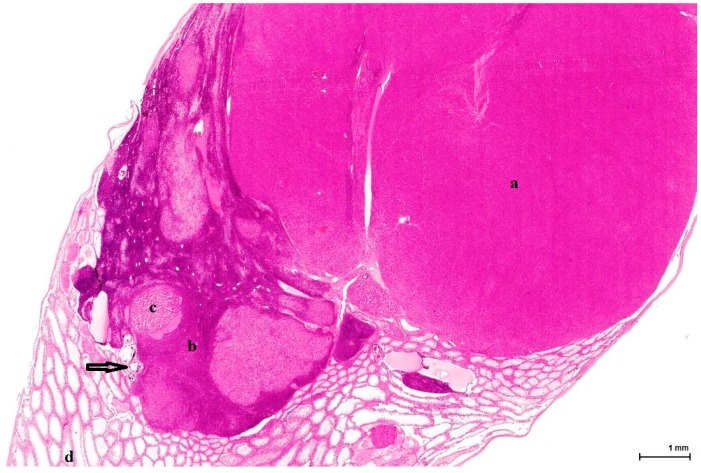
OTA-treated Case 2 with large solid nodule composed of eosinophilic cells (**a**); adjacent sheets of (**b**) basophilic (dark blue) cells including small (**c**) lobules of foamy cells (light eosinophilic). Background: mostly atrophic testicular parenchyma (**d**) with focal calcification (arrow). (H&E).

All cases show enlarged testicles, containing tumours measuring up to 23 mm in the longitudinal sections, well demarcated but not encapsulated, almost completely replacing the whole testis. The tumour invasion front is of pushing type, compressing the minimally remaining testicular parenchyma, which consists of a few normal or atrophic seminiferous tubules, with frequent dystrophic calcification. All tumours are organ-confined; there is no evidence of infiltrative growth or tunica involvement (Figure 1).

**Figure 2:**
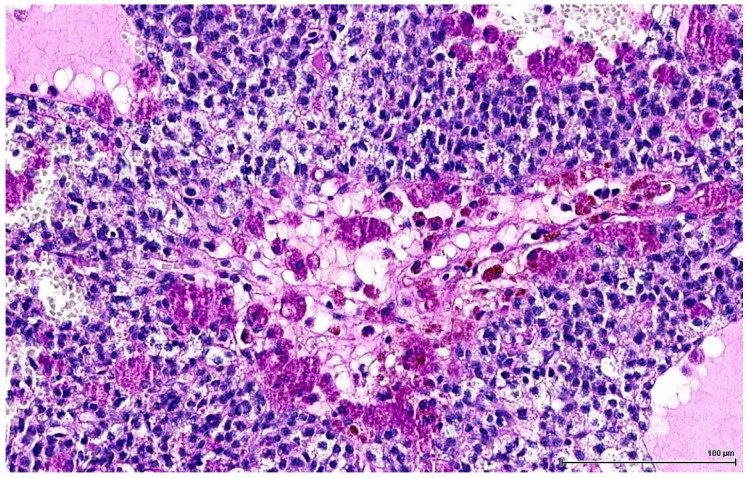
OTA-treated Case 4 showing large cells with foamy cytoplasm containing lipid vacuoles and occasionally magenta/purple lipofuscin pigment (PAS stain).

**Figure 3:**
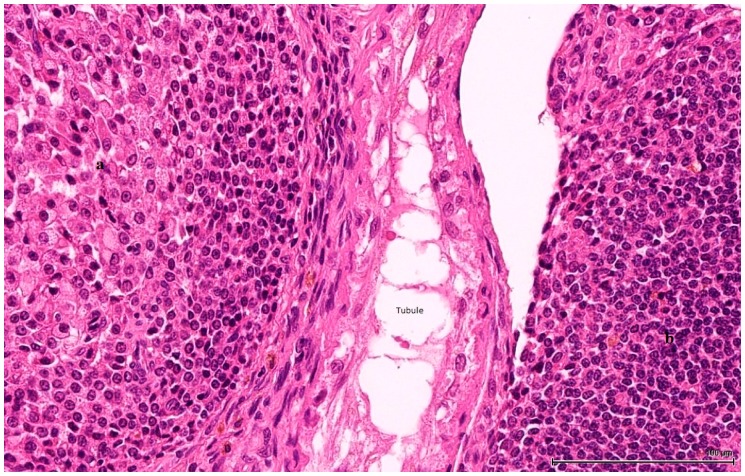
OTA-treated Case 2. Two cell populations of LCT. On the left side: larger cells, with eosinophilic and foamy cytoplasm (**a**) and on the right small cells with basophilic appearance (**b**), mimicking a lymphoid infiltrate. In between, an atrophic seminiferous tubule (H&E).

These are nodular cellular proliferations, usually multinodular, frequently coalescent nodules, usually solid with cystic spaces (empty or filled with proteinaceous material), composed of sheets of large, polyhedral cells with abundant eosinophilic cytoplasm, which is frequently lipidized. The nuclei are uniform, round, with evenly distributed chromatin and occasional small nucleoli; some are mitotically active. Admixed with these large cells are smaller ones, with little, unremarkable cytoplasm and small hyperchromatic nuclei. These two cell populations can form separate individual nodules, or they can intermingle, with no sharp demarcation, as there seems to be an imperceptible transition from one to the other. The large, clear cells with foamy, micro- and macrovesicular cytoplasm contain lipid vacuoles and sometimes golden brown lipofuscin pigment identified as purple red on a PAS stain (Figure 2). The small cell type has a basophilic appearance, mimicking a lymphoid infiltrate (Figure 3). The stroma is scant and occasionally hyalinised, with a rich vascular network; extensive haemorrhaging is present in some cases.

#### 2.1.2. Controls

Findings for control rats are summarized in Table 2.

**Figure 4:**
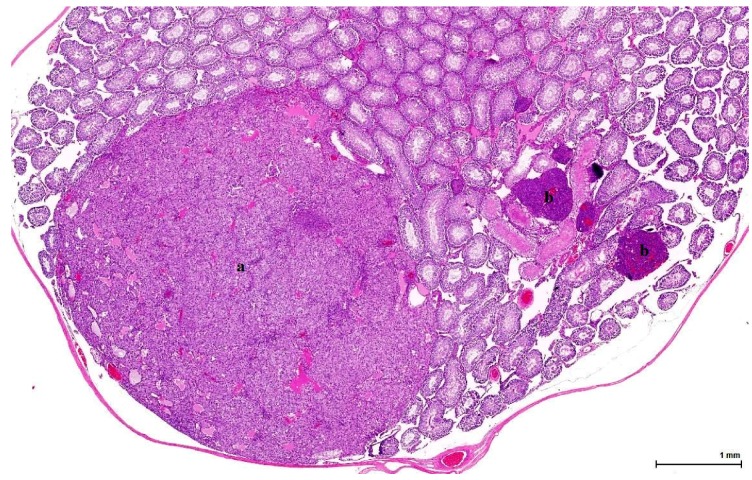
Ginkgo control Case 2 (Table 2). Dominant nodule (**a**) composed of pale eosinophilic cells; on the right (**b**), small nodules of basophilic cells (dark blue), measuring less than the three adjacent tubules (LCH of isolated finding). (H&E).

OTA controls from the 1989 NTP study show the same type of tumour as those exposed daily to OTA for many months in London in the 2000s. Disease was bilateral in 5/6 cases and in the standard transverse sections measured up to 13 mm, with one being larger than the other. The remaining testicular parenchyma is either normal, with active spermatogenesis, or atrophic, with calcifications within a few enlarged tubules. The non-tumoral section shows a few nodules of small basophilic interstitial cells, not distorting the normal architecture and measuring less than the three adjacent tubules (Figure 4).

In controls from the Ginkgo study, half of the cases show tumour on both slide sections (50% bilateral disease), either as one compact nodule or as multiple isolated or coalescent nodules, with solid and cystic architecture, placed in a normal or atrophic testis, with focal intratubular calcifications. The other half of the cases shows the same type of tumour, but on one section only (unilateral disease); the other section (non-tumoral) shows only a few nodules of interstitial cells between normal seminiferous tubules.

The tumour cell types are identical in all cases, with the general impression that the cytoplasm of the large cell component in controls is not as extensively vacuolated as in tumours of OTA-treated rats, being eosinophilic rather than foamy and clear.

### 2.2. Immunohistochemistry

All OTA-treated cases are PLAP- and OCT3/4-negative (Table 1); additionally, two of them (cases 2 and 5) are also negative for D2-40 and CD117. This immune profile rules out a germinal cell tumour. The tumour cells, including the small cell basophilic component, are negative for CD45, CD20 and CD3 (case 4), thus excluding the lymphoid nature of the tumour.

**Figure 5:**
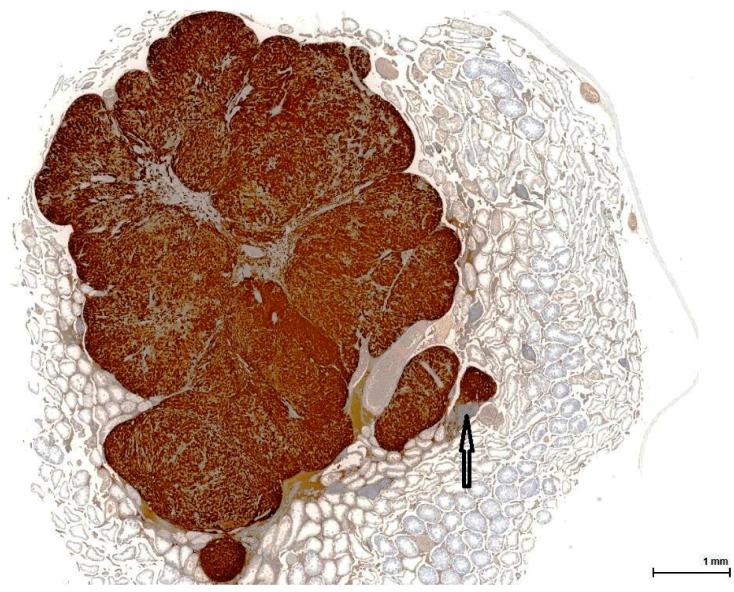
Control rat testis from NTP study on OTA (Case 6, Table 2). Intense and diffuse positive immune reaction (brown) with small cell negative area (arrow) (Calretinin).

**Figure 6:**
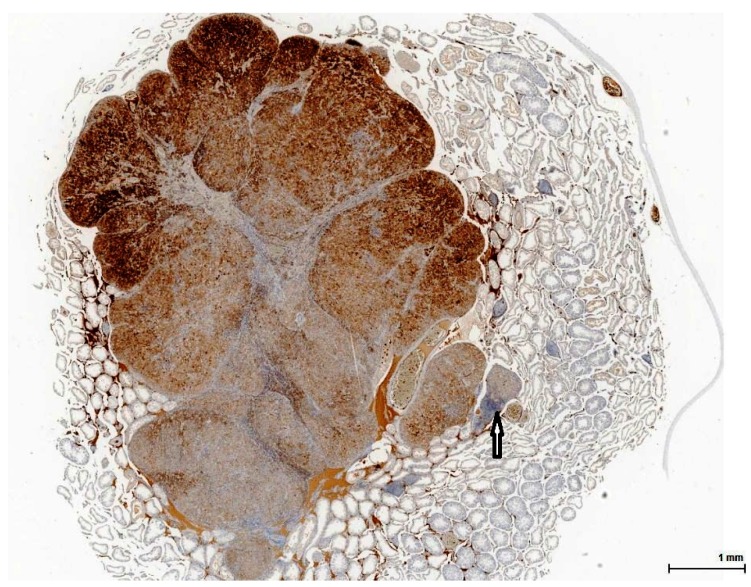
Control rat testis from NTP study on OTA (Case 6, Table 2). Tissue section adjacent to that in Figure 5. Variably positive immune reaction for Melan A (brown) with small cell negative area (arrow) (Melan A).

The OTA-treated cases as well as the controls are positive for Calretinin (Figure 5) and Inhibin A to various degrees and in different distributions. This immune profile is supportive of sex cord-stromal origin; the positive immune reaction for Melan A (Figure 6) is suggestive of a Leydig cell tumour.

**Figure 7:**
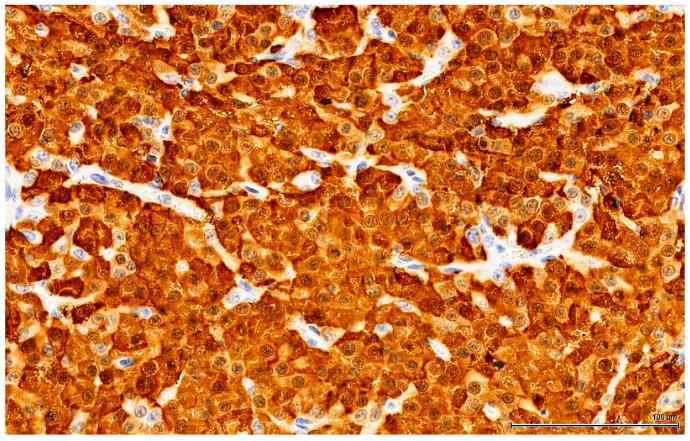
OTA-treated Case 2. Intense and diffuse positive immune reaction (Calretinin).

Calretinin is a calcium-binding protein expressed by a large range of cells or tissues. In the testis, Sertoli cells and Leydig cells are positive, as well as the rete testis epithelium; in clinical practice it is used mostly to establish the mesothelial origin of a pleural tumour (mesothelioma). In our study, the majority of large neoplastic cells (OTA-treated and controls alike) show a diffuse and intense immune staining of the cytoplasm (fine, vesicular): the small basophilic cell component is rather negative, while the intermediate cells are only weakly and inconstantly positive (Figure 7).

**Figure 8:**
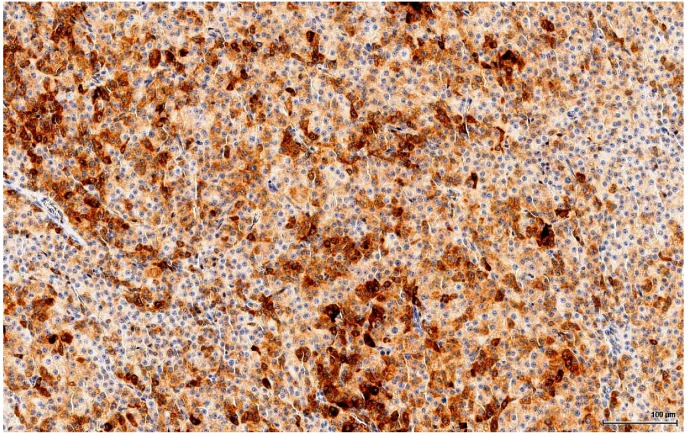
OTA-treated Case 2. Positive immune reaction with variable intensity from strong to weak, focally intense in individual cells (Inhibin A).

Inhibin A is a dimeric glycoprotein that has greater specificity than Calretinin, but the latter has a greater sensitivity for sex cord-stromal tumours; it is expressed by ovarian and testicular normal structures and tumours, including Sertoli and Leydig cells and their corresponding tumours. In this study, the neoplastic cells have a finely granular cytoplasmic immune reaction pattern with membranal accentuation for Inhibin A areas of positive cells, with variable intensity from strong to weak, with focal intense positive reaction in isolated, individual cells. In large cells, there is strong and moderate expression; the small cell population is usually negative. The staining pattern is similar for both OTA-treated and controls (Figure 8).

**Figure 9:**
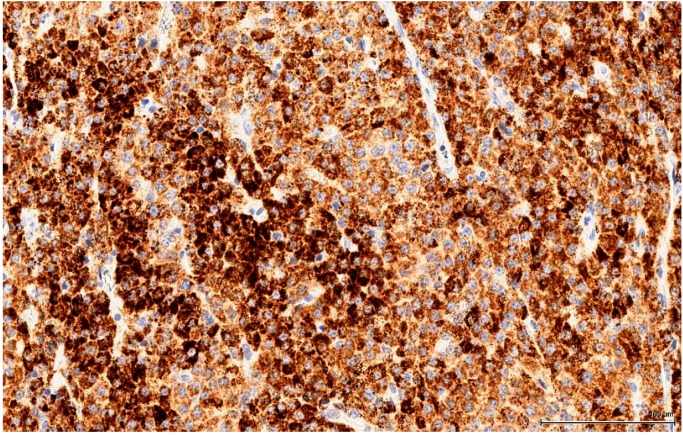
OTA-treated Case 2. Granular positive immune reaction, moderate and intense (Melan A).

Melan A is a melanocyte-specific cytoplasmic protein involved in the melanosome formation of the skin. In the ovary and testis, it is positive in steroid-producing cells due to antibody cross-reactivity to an unknown molecule in these cells; these cells do not produce Melan A. The large neoplastic cells of OTA-treated rat tumours show areas of strong granular cytoplasmic staining reaction (Figure 9), but negative areas as well, especially composed of small cells. In controls, the immune reaction is weaker to negative in two cases, present sometimes with granular pattern in the large cells component, and focally stronger in small cell areas.

## 3. Discussion

It may first be appropriate to reiterate the historic origin of Leydig cell (LC), as: “From the comparative histology of the testis it is clear that, in addition to the seminiferous tubules, blood vessels, and nerves, one finds an additional constant component in the mammalian testis, namely a cell-like mass that when present in smaller amount follows the course of blood vessels between the seminiferous tubules, but when more developed, becomes a mass in which the seminiferous tubules are embedded. Its main constituents are small granules of fatty appearance, which are unaltered by acetic acid and sodium hydroxide treatment, are colourless or yellowish, and encompasses clear, bubble-like vesicular nuclei. Its semifluid ground substance may condense into a cell membrane, and at least in some mammalians the entire granular mass is surrounded by a sharp outline. Also, at times the entire structural aggregate is of such an appearance that one can speak of it as a complete cell” [17].

In more modern terms: LC is a testicular interstitial cell with abundant eosinophilic (rich in smooth endoplasmic reticulum) vesicular (lipid droplets) cytoplasm and a round nucleus, which resides between seminiferous tubules in small groups. It has a tremendous role in testosterone production under luteinizing hormone control, with major implications in spermatogenesis and male secondary sexual characteristics. However, this endocrine function of LC was initially disregarded; only a century later did direct evidence of androgen production emerge by means of histochemistry (1958) and biochemistry (1965) [18].

Laboratory rats appear to be particularly predisposed to Leydig cell hyperplasia (LCH) and Leydig cell tumour (LCT) formation, both spontaneously (increasing with age) and in response to the administration of various xenobiotics [19].

### 3.1. Diagnosis: Hyperplasia Versus Tumour

The distinction between Leydig cell hyperplasia (LCH) and the benign tumour LC adenoma is based on size, because the cytological features can not distinguish between them; the cells look identical, and no morphological criterion can be reliably used to differentiate between them. It was the U.S. National Toxicology Program (NTP) who recommended using the size of adjacent tubules as a threshold. Initially, the cutoff point was 1 (one), so that when the lesion grows larger than the diameter of the adjacent tubule it is diagnosed as adenoma. In 2005 toxicological pathology societies tried to standardize the nomenclature for proliferative and non-proliferative lesions in rats and mice. This initiative is termed the International Harmonization of Nomenclature and Diagnostic Criteria for Lesions in Rats and Mice (INHAND), focusing on specific organ systems. For the male reproductive system, INHAND guidance differentiates hyperplasia from adenoma using a size of three (three) seminiferous tubules, among other criteria [20]. In addition it was suggested that focal hyperplasia should comprise well-differentiated cells devoid of significant mitotic activity, or endocrine sinusoidal network and no evidence of coalescence with adjacent nodules [19].

Histologically, LCH may be diffuse, focal or multifocal; the constituent cells are cytologically unremarkable, showing neither marked changes in size, nor pleomorphic or mitotically active nuclei [21]. This lesion has no expansive growth and does not distort or compress adjacent parenchyma. Based on this axiom, LCH was diagnosed as an accompanying lesion in only one OTA control case and in half of the Ginkgo control cases as small nodules, measuring fewer than three adjacent tubules, usually multiple, in an otherwise normal (three cases) or atrophic (one case) testis. By contrast, adenomas were diagnosed in all cases (OTA and both controls) as large, expansile masses, solitary or multiple.

The malignant counterpart of LC adenoma is rare (10% of LCTs) and is diagnosed upon classical criteria for malignancy: infiltrative growth pattern and cytological features in keeping with cancer (nuclear pleomorphism, atypical mitotic figures). No malignant characteristics were noted in the examined sections.

### 3.2. Differential Diagnosis 

The clear, glycogen-rich, PAS positive cytoplasm of seminoma cells can mimic the clear, lipid-rich cytoplasm of large, foamy LCs [22]. This is an important distinction to make because there is a significant difference in terms of the therapy approach. Immunohistochemistry plays a huge role here, discriminating with certainty between seminoma (a germinal cell tumour) and LCT (a sex cord-stromal tumour), which has the opposite immune profile. In our study, seminoma was ruled out by the absent reaction for germ cell markers (OCT3/4, PLAP) and D2-40 & CD117 (usually positive in seminoma).

Likewise, another important differential diagnosis is lymphoid proliferation, either diffuse or with pseudo-follicular architecture, generating the image of active germinal centres (clear, large cells), surrounded by a mantle of smaller cells. However, the specific type of invasion, diffuse intertubular infiltration [21], is not encountered in our cases. Fortunately, this disease can be easily ruled out by immunophenotyping lymphomas, using cluster of differentiation (CD) molecules, like CD45, CD20 and CD3 (negative on our LCTs).

More subtle differentiation can be problematic between two types of sex cord-stromal tumours: a Sertoli cell tumour of solid type with foamy cells can resemble on H&E a LCT with extensive clear cell change. Even immunohistochemically they share the same immune profile, except that Melan A, which discriminates between these two entities, is positive in LCTs. Notably, Inhibin A consistently stains LCTs, but is also positive in 30‒80% of Sertoli cell tumours [21] and thus cannot be reliably used as a discriminating factor. Rarely, there are also mixed sex cord-stromal tumours composed of Sertoli cells and LCs.

Finally, mesothelioma can be ruled out on our sections by location (no relation to the tunica). However, mesothelial cells can be morphologically versatile and mimic theoretically any cell type, including LC. Furthermore, mesothelial cells are also Calretinin positive, but the complete immune profile (Inhibin A and Melan A) points towards a sex cord-stromal cell origin. None of the tumours examined in this study was accompanied by any form of proliferation of the mesothelial layer of the tunica; at most, a focal minor papillary hyperplastic reaction was noticed. 

### 3.3. Histology Recapitulates the Evolution of Leydig Cells

Differentiation of the rat LC can be broken down into four stages: stem cell, progenitor cell, immature and adult (mature) LC. Stem LCs are spindle-shaped and, as they have not yet committed to a lineage of development, do not express LC-specific markers. At postnatal day 14, stem cells begin to express these markers and are identified at this point as progenitor LCs. These cells develop from about postnatal day 14 until day 28 and begin to produce androgen. Starting at postnatal day 28, progenitor cells transform morphologically to become more round and are termed immature LCs. The immature population doubles once from postnatal day 28 to 56, at which point they develop into adult (mature) LCs. The androgen-metabolizing enzyme activity reduces, while the synthesis of testosterone increases. By day 90, testosterone production in an adult LC of a rat is 150 times that of a progenitor, and five times that of an immature LC [20].

In adult rodents, normal LCs vary morphologically: they are typically round and contain large amounts of eosinophilic cytoplasm, but can also be spindle-shaped with little cytoplasm, with a basophilic appearance. Hyperplasia of these cells is characterized by aggregates of cells that expand the interstitium between seminiferous tubules; hyperplastic LCs are either large, round, with centrally located nuclei and abundant, granular, eosinophilic cytoplasm that becomes vesicular and clear, or, more frequently, smaller, spindle-shaped cells with little cytoplasm and dark, hyperchromatic nuclei. LC adenoma presumably begins as a hyperplastic lesion and consists of one or more expansile foci of LCs that typically compress adjacent tubules: benign Leydig tumour cells are essentially the same size and shape as those encountered in hyperplasia; the distinction between hyperplasia and adenoma is made upon size (three adjacent tubules). These hyperplastic small nodular foci are composed of LCs, which are frequently basophilic in staining characteristics, and it has been suggested that they represent the earliest stages of LCT in the older rat [19].

In the light of the above, the morphologically different cell populations noted in the present study might actually represent various ontogeny stages. The larger, eosinophilic cells could be identified with the more mature stages of differentiation and the smaller, basophilic cells could be assimilated to the more primitive stem and progenitor stages. In between, there are some cells with intermediate morphological features that could represent transition moments between stages. The immune profile of these different cell populations is supportive of this theory: the less differentiated LC (stem and progenitor) do not express the diagnostic markers (Calretinin, Inhibin A and Melan A) in the same way as the more mature typical LC; usually the immune reaction is absent or only focally present in these primitive cells. Furthermore, the intermediate cells appear to be more differentiated and express these markers, but not as robust as the mature LCs. 

### 3.4. Comparison Between LCTs in Rodents and Humans

The incidence of LCTs in rodents increases with age and is highly dependent on species and strain: it ranges from nearly 100% in Fisher 344 rats to 5% in Wistar rats and 6.5% for Sprague‒Dawley rats [20]. In control rats from two-year toxicology and carcinogenicity studies, the incidence of LCTs is 88%, while in life span studies it is up to 96% [23]. Notably, in Fischer 344 rats LCH occurs in quite young animals, appearing to represent a stage in the progression toward LC neoplasms. In humans LCTs are rare and found in all ages, from prepubertal boys to older men, and the incidence is markedly lower than in rodents, making LCT about 0.01% of all cancers in men. Microscopically, the human tumoral LCs resemble those in rodents except for the presence of Reinke crystals, absent in rodents [20].

The high incidence of LCTs in F 344 rats represents a problem in carcinogenicity studies. This strain was used by the NTP for over five decades for toxicity and carcinogenicity studies. However, in 2006, the NTP switched to a different rat stock, largely due to high background control incidences of LCTs with associated F344-specific tunica vaginalis mesothelioma and mononuclear cell leukaemia. The high spontaneous incidence of LCTs in the testes of male F344 rats has made this tumour endpoint of little practical use in identifying potential testicular carcinogenic responses. Therefore, this strain is now abandoned in NTP carcinogenicity studies because it is not appropriate for human health risk assessment and lacks relevance in predicting human carcinogenicity. Initially F 344 was briefly replaced by the Wistar strain, and finally by Sprague‒Dawley [24].

### 3.5. Ochratoxin A as a Putative Risk Factor for Human Testicular Cancer

Rapid dissemination of the ‘OTA as a testicular carcinogen’ hypothesis [9] followed via Lancet Oncology [25], in which there was confusion over rats and mice concerning DNA adducts, and a recommendation that “scientists should not dismiss the theory.” Another recommendation that “further studies could be done with animals” ignored the findings of the comprehensive NTP study 20 years before. Nevertheless, the essence of [9] was mentioned correctly in a popular booklet on testicular cancer [26]. Unfortunately, however, a clear diagnosis of natural testicular tumours as Leydig cell tumours in ageing Fischer rats, and their occurrence irrespective of sexual history [27], was ignored.

Subsequently, reiteration of [13] in [28] perpetuated the focus on the equivalence of “germ cell testicular tumours in mice and humans,” without any corroborative histological evidence. More recently, analysis for OTA in archived blood plasma and testicular germ cell human tumours [29] found traces of the toxin in plasma but not in tumour histology. That finding does not take into account that histology solvents would remove OTA. Otherwise, with a suitable technique, OTA might have been found in any vascularised tissue (e.g., testicular tumours) [30], but that would only demonstrate the common trace of OTA in some human diets well below any verified risk to health.

Literature reviews can also perpetuate errors and misunderstandings. For example, [31] repeats the mistaken conclusion from [7] that the reported testicular tumours were an abnormal occurrence in older Fischer rats. They were never claimed to have anything to do with the OTA exposure and were mentioned purely to illustrate the pathological context (renal pathology for the present cases) (Table 1). Also, a citation from [16] concerning testicular cancer predicted in mice ignores the first major lifetime high-dose-OTA/rodent pathology study [1], demonstrating renal tumours in male mice but making no mention of any testicular lesions. Schwartz et al. [16] had also cited support from the literature on germ cell tumours, teratomas and Sertoli cells, which are not part of the current OTA pathology considerations, which inevitably focus on sex cord-stromal tumours.

Hence, the assertion in [31] that “OTA may be causally related to germ cell testicular tumours in mice and men” and “is a biologically plausible cause of testicular cancer in man” is not supported by experimental animal pathology. The present review of testicular lesion pathology in rats, given many months of continuous OTA intake, shows the tumour cytology to be identical to that occurring naturally. It seems that the male Fischer rat, with its Leydig cell tumours, is also not a legitimate OTA model for predicting the aetiology of the germ cell testicular tumours that account for most testicular cancers in men.

## 4. Methods

### 4.1. Sources of Animal Tissue

Nine tumorous testes were sourced from archived material from several Fischer F344 rat lifetime studies in London during the 2000s ([4,5,7]; UK Home Office licence PPL 70/4720, accessed on 13 January 1999). They relate to necropsy findings during an otherwise specific focus on kidney cancer caused during protracted dietary exposure to OTA. The circumstances sometimes allowed for attention to abnormal (tumorous) testes, which were then fixed in case of subsequent histological interest. Since the testes of untreated controls had not warranted similar attention, a request has been made to NTP Archives for transverse sections of six tumorous testes, mounted on charged slides, from controls of the 1989 NTP study of OTA. In case there was any subsequent long-term genome shift in Fischer rats in the USA, we also requested six analogous examples from controls for the 2013 study on *Ginkgo biloba* extract. All NTP slides for control rats bore a section of both testes.

### 4.2. Immunohistochemical Preparation

Immunohistochemical staining of 3-μm sections (mainly longitudinal) on charged slides (TOMO, Matsunami, Japan) was performed in the Cell Pathology Laboratory of South West London Pathology at St George’s Hospital, Tooting, variously applying panels of antibodies by Roche fully-automated BenchMark ULTRA immunohistochemistry processing as required to obtain clinical diagnoses. The following antibodies (clone) were used: 

Calretinin (SP65), Inhibin A (R1), Melan A (MART1[A103]), OCT3/4 (N1NK), PLAP (NB10), D2-40 (Podoplanin), CD3(2GV6), CD 20 (L26), CD117(C-KIT Polyclonal), CD45(2B11). Each antibody was provided by the supplier with its own data sheet, indicating the clone, concentration and working protocol.

Haematoxylin & eosin and periodic acid schiff staining was also performed for preliminary standard tissue differentiation of nuclear (blue) and cytoplasmic components (red), and glycogen, some mucins and inclusions (magenta), respectively.

## Figures and Tables

**Table 1:** Ochratoxin A exposure context for rat testicular lesion immunohistochemistry, showing toxin dosage, age at death, renal cancer incidence, and testicular tumour recognition by histology. Immunohistochemical profile by PLAP and OCT3/4 negative immunostaining excludes germinal cell diagnosis. Responses to Calretinin, Inhibin and Melan A antibody staining confirm diagnosis of Leydig cell tumours. * [3], ** [4], *** [7].

Case	OTA Dose Daily (µg/kg b.w.)		Duration	Age at Death, Days	Renal Cancer	Testis Pathology (H and E)	Immunohistochemical Staining			
							PLAP and OCT3/4	Calretinin	Inhibin	Melan A
1	300	*	10 months in 1st year	750	No	tumour	-	+++	++	+++
2	50	**	Lifetime	660	Yes	tumour	-	+++	++	+++
3	50	**	Lifetime	750	Yes	tumour	-	+++	++	+
4	50	**	Lifetime	780	Yes	tumour	-	Not tested		
5	300	***	9 months in 2nd year	690	No	tumour	-	+++	++	++
6	300	***	10 months in 2nd year	720	No	tumour	-	+++	+	-
7	300	***	11 months in 2nd year	750	No	tumour	-	+++	+++	+
8	300	***	13 months from 1st year	810	No	tumour	-	+++	+++	++
9	300	***	14 months from 1st year	840	Yes	tumour	-	+++	+	+

**Table 2:** Comparative summary of immunohistochemistry responses to Calretinin, Inhibin and Melan A in testicular tumours of F 344 control rats from NTP lifetime studies on ochratoxin A and *Ginkgo biloba* extract, in the context of the slight age difference at death.

**NTP OTA Controls**	**Calretinin**	**Inhibin**	**Melan A**	**Age at Death (Days)**
1	+++	++	+	792
2	+++	++	+	793
3	+++	++	++	793
4	+++	++	++	775
5	+++	+	++	794
6	+++	+	++	767
**NTP GINKGO Controls**	**Calretinin**	**Inhibin**	**Melan A**	**Age at Death (Days)**
1	+++	+	+	745
2	+++	++	negative	774
3	+++	++	negative	774
4	+++	++	+	774
5	+++	++	+	773
6	+++	++	+	775
